# Screening and monitoring of diabetic polyneuropathy in clinical practice: present and future with connected devices

**DOI:** 10.3389/fneur.2025.1679277

**Published:** 2025-10-21

**Authors:** Jean-Pascal Lefaucheur

**Affiliations:** ^1^Unité de Neurophysiologie Clinique, Hôpital Henri Mondor, AP-HP, Créteil, France; ^2^EA4391 (ENT), Faculté de Santé, Université Paris Est Créteil, Créteil, France

**Keywords:** autonomic neuropathy, diabetes, diagnosis, monitoring, polyneuropathy, small fiber neuropathy, smartphone, telemedicine

## Abstract

New perspectives are opening up today in the management of diabetes thanks to the possibility of measuring, over long periods in daily life, different biomarkers likely to improve glycaemic control, such as continuous glucose monitoring and time-in-range assessment. This is part of personalized medicine. There is therefore a challenge to also benefit from specific biomarkers in the prevention and monitoring of polyneuropathy in diabetics, one of the most common type of peripheral nerve disorder worldwide. This is now possible with the development of connected tools, allowing for example to monitor at home the evolution of skin temperature or conductance at the level of the feet. In this article, the current use and recent advances in laboratory tools for the early diagnosis and objective monitoring of diabetic polyneuropathy and its progression will be presented. The follow-up of neuropathies will undoubtedly be significantly modified in clinical practice in the future, particularly in the context of diabetes, thanks to the use of connected tools and remote monitoring.

## Introduction

Diabetic neuropathy (DN) affects millions of people worldwide, impairing quality of life and daily functioning (1, 2). DN is also associated with an increased relative risk of death, especially due to the dysfunction of the peripheral autonomic nervous system (3, 4). This highly morbid disorder is therefore the cause of major socio-economic problems and very significant annual health costs, even only considering the diabetic foot syndrome, a dramatic consequence leading to difficult-to-treat ulcers and amputations (5, 6). Diabetic foot syndrome is defined by the World Health Organization as an “ulceration of the foot (distally from the ankle and including the ankle) associated with neuropathy and different grades of ischemia and infection.”

Distal symmetric polyneuropathy (DPN) is the most common form of DN, characterized by the progressive damage and loss of various populations of nerve fibers in a symmetrical and length-dependent pattern, therefore starting at the feet (7–10). The clinical picture includes a variable mix of negative sensory signs and symptoms (hypoesthesia and numbness) and positive sensory signs and symptoms (non-painful paresthesias, such as tingling, or painful dysesthesias, whether spontaneous or evoked). These sensory features involve large-diameter A-beta nerve fibers and small-diameter A-delta and type C nerve fibers. Unmyelinated C fibers are also involved in the autonomic part of DPN, mainly at the origin of vasomotor or sudomotor dysfunction of the limb extremities (11, 12). In more advanced cases of DPN, this can result in ulcers, infections and amputations in the feet, as well as loss or dysfunction of larger-diameter nerve fibers involved in motor or proprioceptive function. Ultimately, the patients may show balance disorders and an increased risk of falls, unnoticed injuries, and fractures (13, 14).

Also, to avoid this deleterious evolution and to prevent morbidity and complications, there is an obvious need to develop laboratory tools allowing DPN to be diagnosed early, especially because of a frequent asymptomatic onset (15), and also to objectively monitor its evolution. These tools could be directed towards the detection of neurodegeneration, for example by measuring serum neurofilament light chains (sNfL) levels (16–18). However, the value of sNfL measurement has been shown to be neither sensitive (19) nor specific (with respect to the detection of central nervous system involvement) for the diagnosis of DPN (20). Thus, from a more neurophysiological perspective, these tools must be more specifically linked to the evaluation of a given type of nerve fibers at the level of the feet, repeatable, reproducible, and sensitive to alterations and early changes in nerve function.

### Different assessment tools for different nerve fiber types

**Figure 1:**
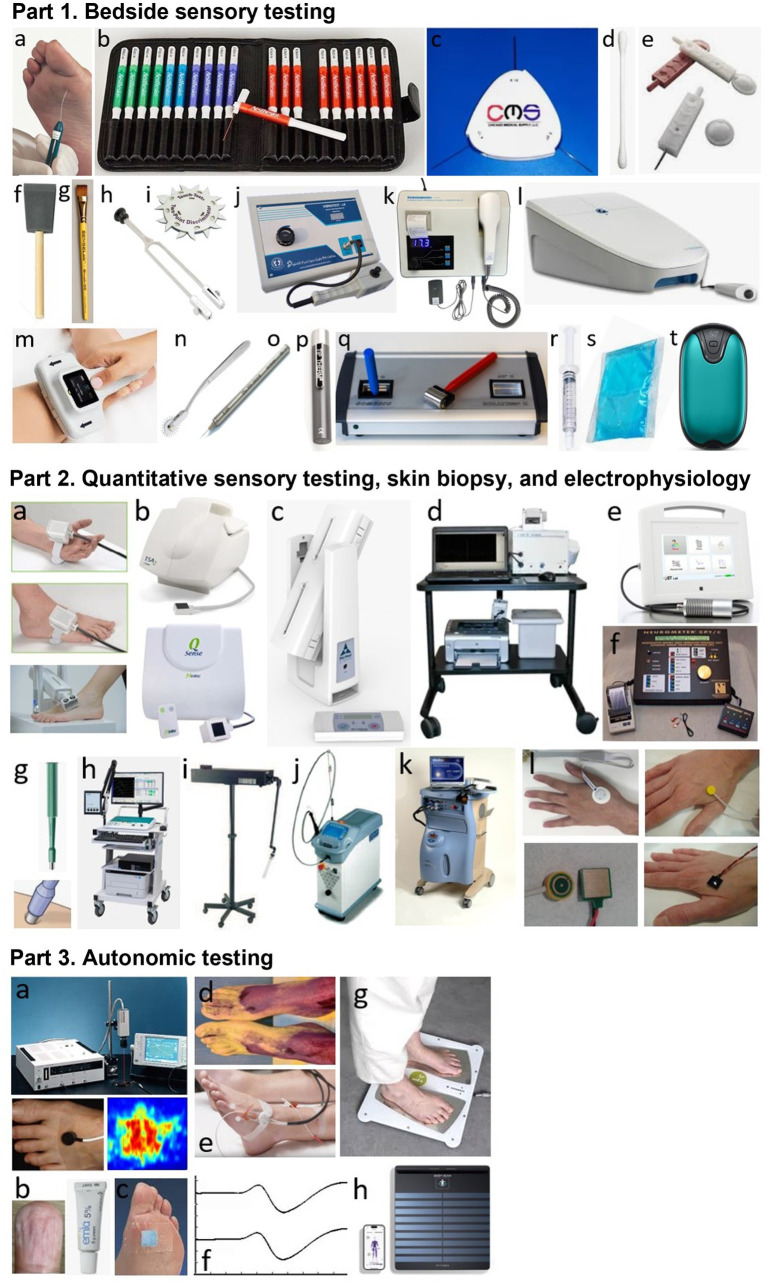
Part 1. Bedside sensory testing. a: 10-g monofilament, b: von Frey/Semmes-Weinstein monofilaments testing kit, c: 1/10/75-g nylon filament wheel, d: Q-tip, e: sharp and round tips of Neurotip^®^, f: foam brush, g: calibrated hair brush, h: 128-Hz (Rydel-Seiffer) tuning fork, i: two-point discriminator wheel, j: Vibrometer^®^, k: Biothesiometer^®^, l: VibroSense^®^, m: DPNCheck®, n: Wartenberg wheel, o: Pin prick^®^ stimulator, p: Tip Therm^®^, q: Rolltemp^®^, r: filled syringe, s: cooling pack, t: digital hand warmer. Part 2. Quantitative sensory testing, skin biopsy, and electrophysiology. a: thermodes, b: TSA2® and Q-Sense^®^, c: NerveCheck^®^, d: Case IV^®^, e: QST. Lab^®^, f: Neurometer^®^, g: disposable skin biopsy punch, h; machine for performing nerve conduction study or evoked potentials, i: CO2 laser, j: Nd: YAP laser, k; contact-heat evoked potentials, l: different electrodes for performing intraepidermal evoked potentials. Part 3. Autonomic testing (at foot level). a: Laser doppler flowmetry or imaging, b: EMLA test, c: Neuropad^®^, d: thermoregulatory sweat test, e: QSART^®^, f: sympathetic skin response, g: Sudoscan^®^, h: Body Scan^®^.

Mainly four types of nerve fibers must be assessed: A-beta sensory fibers, A-delta sensory fibers, type C sensory fibers, and type C autonomic fibers. The various tests that can be used in clinical practice to assess impairment of these different types of nerve fibers in the feet are presented in Table 1 and Figure 1.

**Table 1:** Assessment tools according to the type of peripheral nerve fibers.

Type of assessment tool	A-beta sensory nerve fibers	A-delta sensory nerve fibers	C sensory nerve fibers	C autonomic nerve fibers
10-g monofilament	Light-touch pressure			
von Frey / Semmes-Weinstein monofilaments testing kit (1/10/75-g nylon filament wheel)	Light-touch pressure			
Q-tip, round tip of Neurotip^®^	Light-touch pressure			
Foam / hair brush	Light-touch pressure			
Two-point discriminator wheel	Light-touch pressure			
128-Hz (Rydel-Seiffer) tuning fork	Vibratory sensation			
Vibrometer^®^, Biothesiometer^®^, Neurothesiometer^®^, VibroSense^®^	Vibration detection threshold (VDT)			
Sensory nerve conduction study (eg, DPNCheck^®^)	Sensory nerve action potential (SNAP) amplitude and velocity			
Safety pin (eg, Neurotip^®^ combined with a Neuropen^®^)		Pinprick sensation		
Wartenberg wheel		Pinprick sensation		
Pin prick® stimulators testing kit		Pinprick sensation		
Metal rods/rollers (eg, Tip Therm^®^, Rolltemp^®^)		Cold temperature sensation	Warm temperature sensation	
Syringe with frozen/warm liquid		Cold temperature sensation	Warm temperature sensation	
Cooling pack, digital hand warmer		Cold temperature sensation	Warm temperature sensation	
Quantitative sensory testing machine (NerveCheck^®^, TSA2^®^, Q-Sense^®^, Case IV^®^, QST Lab^®^)	Vibration detection threshold (VDT)	Cold detection threshold (CDT)	Warm/heat pain detection threshold (WDT, HPT)	
Current perception threshold (CPT, Neurometer^®^)	CPT at 2000 Hz	CPT at 250 Hz	CPT at 5 Hz	
Skin biopsy		Intraepidermal nerve fiber density (IENFD)	Intraepidermal nerve fiber density (IENFD)	
Somatosensory evoked potentials (SSEPs)	SSEP amplitude and latency			
Laser/intraepidermal/contact-heat evoked potentials (LEPs, IEEPs, CHEPs)		LEP/IEEP amplitude and latency	CHEP amplitude and latency	
Laser doppler flowmetry or imaging				Flow or flare measurement
EMLA test				Skin wrinkling measurement
Thermoregulatory sweat test				Color change assessment
Neuropad^®^				Color change assessment
Quantitative sudomotor axon reflex test (QSART^®^)				Sweat response measurement
Sympathetic skin response (SSR)				SSR amplitude and latency
Electrochemical skin conductance (ESC, Sudoscan^®^, Body Scan^®^)				ESC measurement

In routine practice, the screening of DPN is based on the assessment of large A-beta fibers involved in light touch on three or four plantar sites with a 10-g monofilament and involved in vibration sense on the dorsal aspect of the great toe (interphalangeal joint) with a 128-Hz tuning fork (10). Other simple tools can be used for bedside sensory testing, such as the two-point discrimination test (21), which appears to measure sensory properties of the foot that differ from light touch assessed using monofilaments in diabetic patients (22). In addition, small A-delta fibers can be assessed for pinprick sensation with a safety pin (e.g., Neurotip^®^ combined with a Neuropen^®^) (23) and for cold temperature sensation with a cold metal object (e.g., Tip Therm^®^) (24).

On the other hand, more complex quantitative sensory testing (QST) can be performed using computerized devices (Table 1; Figure 1). These devices allow sensory thresholds to be quantified as numerical values, more accurately than with conventional bedside testing, which is usually performed in a binary manner (stimulation perceived or not). Some tools are of intermediate use and combine portability (portable devices) with quantification of sensory thresholds. This is the case of the Biothesiometer^®^ or Neurothesiometer^®^ to assess vibration detection threshold (25, 26) or the NerveCheck^®^, which also assesses cold, warm, and heat pain detection thresholds with simple paradigms (27, 28).

Sensory nerve fibers can also be assessed using electrophysiological techniques of nerve conduction studies (29, 30). In the context of length-dependent diabetic polyneuropathy, sensory nerve action potentials (SNAPs) should be recorded distally in the lower limbs, particularly for the sural nerves. These recordings can be performed using a conventional EMG device, in conjunction with motor nerve conduction study in this case, or using dedicated devices, such as the DPNCheck^®^, which is limited to recording SNAPs from the sural nerve to the ankle (31, 32). The measurement of SNAPs is a particularly objective method of assessing large-diameter A-beta sensory nerve fibers in their distal segment, but provides no information on smaller-diameter sensory nerve fibers.

For small-diameter nerve fibers, electrophysiological tests can also be performed routinely, using stimulating devices capable of selectively stimulating this type of nerve fibers (33). The stimulation techniques that can be used for this purpose are based on thermal or electrical stimulation, while the recording of “evoked potentials” is performed using scalp electrodes and based on the averaging of electroencephalographic activities. Thermal stimulation can be radiant heating delivered by a laser or contact heating delivered by a thermode, allowing the recording of laser evoked potentials (LEPs) (34–36) or contact-heat evoked potentials (CHEPs) (37, 38), respectively. Electrical stimulation should aim to deliver a very focal current limited to the epidermis, where only the endings of small diameter nerve fibers are present. Different types of electrodes can be used for this purpose, allowing the recording of intraepidermal evoked potentials (IEEPs) (33). Usual somatosensory evoked potentials (SSEPs), obtained with a large bipolar stimulating electrode (as for SNAP recordings), engage subepidermal endings of large-diameter A-beta sensory fibers. The main limitation of using LEPs, CHEPs, IEEPs for the study of small-diameter A-delta or C fibers is that only brain responses can be recorded with these techniques, which precludes the assessment of a purely peripheral component (unlike SSEPs for large-diameter A-beta fibers) (33).

On the other hand, small-diameter sensory nerve endings can be assessed very specifically in the distal lower limbs by measuring intraepidermal nerve fiber density in a small skin biopsy (39–41). However, the representativeness of the measurement on a skin surface as small as a few mm^2^ is questionable. Furthermore, except in dedicated research studies (42), the repeatability of this invasive technique is limited for routine longitudinal monitoring of patients with DPN, particularly due to the increased risk of healing problems. Another technique to study small-diameter sensory innervation is corneal confocal microscopy, with the measurement of intracorneal nerve fiber density, fiber length, or branching density (43, 44). Although these measures may show significant correlations with the existence of more diffuse DPN (45–47), they do not directly assess innervation at the foot level and this technique is therefore less relevant than others for the specific assessment of diabetic foot syndrome.

Small-diameter nerve fibers also include autonomic fibers. Many tests of the autonomic nervous system are applicable in clinical practice (48). However, some tests do not directly assess distal autonomic innervation at the feet, such as cardiac autonomic function tests (Ewing tests) (49). In contrast, other tests specifically assess distal autonomic nerve fibers, which is highly relevant in the context of DPN, and generally rely on the vasomotor or sudomotor aspects of autonomic innervation of the foot (50, 51). There are methods that are easy to implement, but which nevertheless require a fairly long examination time and provide only a semi-quantitative assessment, such as the visualization of local vasoconstriction produced by the cutaneous application of a eutectic mixture of local anesthetics (EMLA test) (52–55) or the Neuropad® plaster test for sudomotor function (56–60). A better quantified assessment of distal autonomic functions can be achieved using more complex, time-consuming, and expensive techniques, such as laser Doppler techniques measuring vasomotor-mediated axon reflexes in response to different types of local cutaneous stimuli using vasoactive drugs, electrical stimulation, or heating (61). Laser Doppler techniques include laser flowmetry (LDF) (62–70) and flare response imaging (LDI) (71–75), but LDF is characterized by high intra- and inter-individual measurement variability and LDI by the lack of standardized image analysis methods, thus limiting their use in clinical practice.

Regarding the assessment of sudomotor function in the limbs, the quantitative sudomotor axon reflex test (QSART), developed in 1983 (76), has been promoted by its inventors as the gold standard technique (77, 78). This technique is based on the measurement, by a sudorometer, of the sweat response to local acetylcholine iontophoresis. However, the QSART technique requires complex expertise, a temperature- and humidity-controlled environment, and a relatively long examination time. In addition, its diagnostic sensitivity is limited by the high variability and low reproducibility of measures performed in the lower limbs (79, 80). Also, another technique, called Sudoscan^®^, simpler and faster (examination time of 2–3 min) than the QSART, has attracted great interest for quantitatively assessing distal sudomotor autonomic innervation of the extremities in clinical practice. The Sudoscan^®^ technique is based on the principle of chronoamperometry and reverse iontophoresis, with measurement of electrochemical skin conductance (ESC) in microSiemens (μS). The ESC measurement depends on the current induced by the release of chloride ions from the eccrine sweat glands following activation by a low constant current of the sympathetic C fibers innervating these glands (81, 82). The Sudoscan® test has demonstrated its validity in the diagnosis of distal autonomic C-fiber lesion associated with DPN (83–94) or distal polyneuropathies of other causes (95). This technique does not require complex operator training (96) and has completely replaced the recording of sympathetic skin responses (SSRs), which was previously the routine electrodiagnostic test for assessing distal autonomic innervation of the limbs (97, 98). Indeed, SSR recording is poorly reproducible (99, 100) and is not specific to distal innervation by sympathetic C-fibers, as it is influenced by large-fiber sensory afferents and central reflex processing.

### Screening strategy for the early diagnosis of DPN

The risk of developing diabetic foot syndrome and therefore presenting with DPN must be assessed annually in primary care according to international recommendations (9, 101, 102). However, this recommendation faces several difficulties. The first is the absence of a sensitive, objective, and validated strategy for diagnosing early DPN. As stated previously, DPN is routinely screened by semi-objective methods assessing touch, pinprick, and temperature sensations. Binns-Hall et al. showed that the combination of distal investigation of large-diameter sensory fires using the DPNCheck^®^ and small-diameter autonomic fires using the Sudoscan^®^ could be sensitive (95%) and specific (82%) to distinguish between the absence and presence of DPN and risk for diabetic foot syndrome with a strong correlation with clinical questionnaires (103).

However, such a one-stop screening strategy requires a hospital setting and many diabetic patients may encounter difficulties accessing hospital structures due to a lack of supplies or specialized structures. This is the reason why a large-scale project was developed in France to perform Sudoscan^®^ in community health structures, ie more than 400 pharmacies. The measurement of ESC at the feet was combined with the Michigan Neuropathy Screening Instrument (104) with the physical assessment completed by the pharmacist, who was also asked to take eight photographs of the patients’ feet from different angles. All these data (ESC values, MNSI scores, and pictures of the feet) were sent by remote transmission to reference diabetology units for analysis. This study showed that reduced ESC in the feet was highly predictive of diabetic foot syndrome, particularly in cases of asymmetric ESC values or ESC values below 50 μS (unpublished data). A similar project had already been proposed in Canada, but using sural neve conduction measurement with the DPNCheck^®^ in community pharmacies, instead of the ESC as a biomarker of DPN (105). The objective is that the pharmacists use these test results to educate patients on preventing DPN through a better glycaemic control and lifestyle, and improving foot self-care to avoid diabetic foot syndrome.

### New perspectives with connected devices and telemedicine

New perspectives for diabetes monitoring are now opening up thanks to the development of connected tools, also adapted in clinical practice as a means of therapeutic education. This is the case of recent innovations such as continuous glucose monitoring (CGM) and time in range (TIR), which are emerging clinical endpoints for improving glycaemic control (106–110).

A variety of approaches have been proposed and studied to improve the management of diabetes by telemedicine (111–116), including the transmission of biomarkers, such as glycaemia (117) or body mass index (118), or telecoaching to improve lifestyle and promote exercise (119–121), or both (122). A telemonitoring program has already been performed in France (EDUC@DOM study) (123, 124), which combined biomedical data measurement with connected objects used at home, including a scale with impedancemetry, actimeter and blood glucose meter, and interactive educational software programs (with artificial intelligence (AI) algorithms). Compared to standard care, the remote monitoring performed by diabetologists with this telemedicine program over one or 2 years tended to result into a greater reduction of HbA1c levels (123) and was significantly cost-saving on socio-economic grounds (124). However, this program did not provide tools or measures to specifically monitor DPN.

It is now possible to measure ESC at the feet using a connected body scale, called Body Scan^®^. The ESC measurements obtained with the Body Scan^®^ in just 20 s are perfectly consistent with those obtained with the Sudoscan^®^, thus allowing to consider a similar sensitivity and specificity in the diagnosis of distal autonomic neuropathy (125). Moreover, compared to the Sudoscan^®^, the advantage of the Body Scan® is that it allows the recording of ESC on a daily basis, at home, by the patients themselves. The association of this connected tool, more specifically assessing DPN, with other connected tools for assessing glycaemic control, could prove interesting. Indeed, a reduction in TIR and an increase in glycaemic variability revealed by CGM have been associated with progression of DPN and reduced ESC values at the feet measured with the Sudoscan^®^ (126, 127).

On the other hand, ESC asymmetry at the feet > 9.5% was found to have 80% sensitivity and 91% specificity to determine the risk of diabetic foot syndrome (128). Thus, including a valuable biomarker of foot innervation, such as ESC, could be a way to improve the detection and monitoring of DPN, more specifically than the telemedicine strategies previously described. It is therefore tempting to design a large-scale cohort study to determine the adherence to a program of at-home ESC measurements at the feet over a long period of time for the follow-up of diabetic patients and monitoring of DPN, in particular to confirm the predictive value of ESC asymmetry in the development of diabetic foot complication.

A concurrent approach is to monitor foot temperature at home, using an infrared thermometer, a sensor mat, or temperature measuring socks (129). Adherence to this type of monitoring was found to range between 56 and 86% and is even better for socks. When the temperature difference between the feet is greater than 2.2 °C (at the hot spot), the patients are recommended to reduce their daily steps by 50% and notify a healthcare professional or podiatrist as this indicates a significantly increased risk of foot ulcers. Constant monitoring of foot temperature could be combined with plantar pressure measurements using sensors embedded in a wearable insole (130). In one study, it was proposed that patients self-assess the plantar thermal images they took at home using smartphone-based thermography (131). Early detection of diabetic foot complication could benefit from AI for thermographic image analysis in future smartphone apps (132, 133).

Another home-based approach with smartphone-based self-photographs aims to assess the presence or extent of foot ulcers (134, 135) by allowing patients to photograph the plantar surface of their feet unassisted [“foot selfie,” (136)] and transmit these images to a remote server. Wound imaging systems with commercial portable devices have already demonstrated high accuracy (137, 138) and are expected to benefit even more from AI and machine-learning algorithms in the future (139–142) to prevent the development of diabetic foot ulcers.

Finally, a novel smartphone-based home monitoring approach to DPN has recently been reported, including patient self-assessment through large fiber sensory testing, including vibration perception and two-point discrimination assessed with 3D-printed accessories, combined with a clinical neuropathy assessment questionnaire (143). In the context of chemotherapy-induced peripheral neuropathy, another group also proposed a smartphone app for neuropathy monitoring, comprising clinical questionnaires and six functional assessments using smartphone sensors to provide information on neurological functions, such as walking, standing, and dexterity (144, 145). In any case, there are increasing perspectives for the use of smart wearable technologies and various types of sensors integrated into smartphones, socks, insoles, or shoes, for continuous or at-home health monitoring, prevention of diabetic foot ulcers or risk of falls, including AI solutions and deep learning models to improve data analysis (146–150).

## Conclusion

In conclusion, DN, including DPN, remains a major health problem, with serious consequences such as diabetic foot syndrome. Early and accurate detection of DPN, particularly through specific and sensitive tools targeting different nerve fiber types, is essential for its prevention and improvement of outcomes. Technological advances, notably through connected devices specifically assessing foot innervation by conductance or temperature measurements for example, offer promising perspectives for continuous home monitoring of nerve function in large cohorts of patients. Combined with connected glucose control measures, telemedicine, and patient education, these innovations could significantly transform the management of DPN by improving early diagnosis, disease monitoring, and overall patient care, which could prevent serious complications such as foot ulcers and amputations, reduce healthcare costs, and improve the quality of life of diabetic patients worldwide.

## Data Availability

The original contributions presented in the study are included in the article/supplementary material, further inquiries can be directed to the corresponding author.
